# Regional White Matter Lesion Load in Elderly Patients With Somatic vs. Non‐Somatic Delusional Disorder

**DOI:** 10.1111/psyg.70055

**Published:** 2025-06-05

**Authors:** Robert Christian Wolf, Mike M. Schmitgen, Marie‐Luise Otte, Martin Karner, Roger Pycha, Erwin Kirchler, Nadine Donata Wolf, Markus Huber

**Affiliations:** ^1^ Department of General Psychiatry Center for Psychosocial Medicine, Heidelberg University Heidelberg Germany; ^2^ DZPG, German Center for Mental Health, Partner Site Mannheim/Heidelberg/Ulm (ZIHUb) Heidelberg Germany; ^3^ Department of Radiology General Hospital Bruneck Brunico/Bruneck South Tyrol Italy; ^4^ Department of Psychiatry General Hospital Brixen Bressanone/Brixen South Tyrol Italy; ^5^ Department of Psychiatry General Hospital Bruneck Brunico/Bruneck South Tyrol Italy

**Keywords:** delusional disorders, lesion segmentation, MRI, somatic delusions, white matter lesions

## Abstract

**Objective:**

Delusional disorders (DD) are among the most debilitating mental disorders in the elderly. Persistent monothematic delusions frequently include paranoid and persecutory beliefs, as well as various forms of somatic delusions, including delusions of being infested by pathogens. So far, little is known about the neural correlates of DD. Yet, particularly in elderly patients, white‐matter lesions (WML) are thought to play an important pathophysiological role.

**Methods:**

To investigate regional WML in patients with DD, structural MRI was used, followed by automated lesion segmentation methods to facilitate WML load (WMLL) comparisons between healthy controls (HC, *n* = 28) and patients with distinct types of DD, that is, somatic (*n* = 16) versus non‐somatic DD (*n* = 17). Patients with somatic DD presented with specific delusional content, that is, beliefs of delusional infestation (DI), whereas individuals with non‐somatic DD (non‐DI) showed predominantly paranoid and persecutory content.

**Results:**

Regions with higher WMLL in both DI and non‐DI patients compared to HC included the anterior cingulate and lateral prefrontal regions located in the middle frontal gyrus. Regions with higher WMLL in DI patients versus both HC and non‐DI patients were predominantly located in the sensorimotor areas of the frontal lobe.

**Conclusion:**

The data suggest distinct patterns of regional WMLL in elderly patients with DI versus non‐DI. The anatomical distribution of WMLL supports a neuromechanistic model that emphasises the importance of brain areas that drive the internal bodily focus of somatic delusions versus the externalised cognitive distortions that can be observed in non‐somatic delusions.

## 1 Introduction

Delusional disorders (DD) are psychotic disorders characterised by fixed beliefs as a single symptom, sometimes accompanied by hallucinations related to the delusional theme [1]. Persistent monothematic delusions frequently include paranoid and persecutory beliefs or various forms of somatic delusions [2, 3, 4]. The prevalence of DD in individuals aged > 65 years is estimated at approximately 0.05%, yet this is very likely a gross underestimation given that individuals with DD mostly show poor to absent illness insight that hampers help‐seeking behaviour and prevents timely diagnosis and treatment [5, 6, 7].

A prominent form of delusional disorder somatic type is delusional infestation (DI), also known as delusional parasitosis or Ekbom syndrome [8]. The clinical picture of delusional infestation (DI) involves a strong, fixed delusional belief that the individual is infested with pathogens such as parasites, insects or microscopic organisms. This belief is often accompanied by tactile hallucinations (e.g., the sensation of crawling, biting or stinging on the skin) and excessive preoccupation with bodily sensations. The affected individuals may exhibit repetitive behaviours like skin picking, self‐examination or cleaning rituals, alongside significant functional impairment [9, 10, 11].

The neural mechanisms of DI and other DD are poorly understood at present, one of the reasons for this gap of knowledge being the still very low number of controlled studies going beyond single cases or case studies with few participants [12]. From a clinician's point of view, however, the dearth of knowledge is less surprising given the well‐known illness insight deficits in patients with DD. Nevertheless, previous structural MRI studies in DI provided evidence for regionally abnormal grey matter volume in brain regions involved in source monitoring, multisensory integration and somatic awareness [13, 14]. Such structural abnormalities could contribute to core clinical features of DI, that is, distorted interpretation of bodily sensations and an inability to suppress irrational beliefs related to such sensations. In addition, white matter abnormalities, particularly in regions connecting frontal and parietal regions, have been also highlighted previously [15]. This is consistent with observations from clinical neuroradiology that consistently point toward various degrees of white matter lesions (WML) in DD, particularly in elderly patients [16, 17]. In clinical practice, such WML are attributed to several conditions ranging from older age to various degrees of cerebral atherosclerosis. Yet, the pathophysiological significance of WML in DD is unclear at present. Here, we use structural MRI imaging and a novel technique for WM segmentation [18] to address regional WML load (WMLL) in 1. patients with DI versus healthy controls (HC) and 2. in patients with DI versus patients with non‐somatic DD (non‐DI). We expected to detect higher frontal WML in both DI and non‐DI, and a more prominent involvement of brain regions engaged in sensorimotor integration processes in DI patients compared with both HC and non‐DI patients.

## 2 Methods

### 2.1 Participants

We investigated 38 right‐handed patients presenting with DD according to DSM‐5 criteria. Patients were recruited at the Psychiatric Department of the General Hospital Bruneck/South Tyrol, Italy. We included 16 patients with a delusional disorder, somatic type (DI‐group) and 17 patients with non‐somatic delusional disorders (non‐DI‐group). The healthy control (HC) group included 28 healthy volunteers. The DI‐group consisted of 11 females and 5 males with a mean age of 71.5 (standard deviation, SD = 11.9) years. Mean disease duration was 6.0 years (SD = 9.4). All DI patients received antipsychotic treatment (mean chlorpromazine [CPZ] equivalents = 179.7, SD = 103.7). The non‐DI‐group consisted of 12 females and 5 males with a mean age of 58.8 (SD = 15.2) years. Non‐DI patients presented with the following non‐somatic delusional content: paranoid and persecutory beliefs only (*n* = 8), mixed persecution/poisoning (*n* = 5), jealousy (*n* = 2, without any association to any substance‐use disorder) and poverty (*n* = 2). Mean disease duration was 13.5 years (SD = 10.5). Three patients were unmedicated. Fourteen patients received antipsychotic treatment (mean chlorpromazine [CPZ] equivalents = 171.3, SD = 96.4). None of the DD patients had a history of substance‐use disorder or showed clinical signs of dementia, and clinical brain imaging and laboratory work‐up were not suggestive of an underlying medical condition causing DD. The healthy control (HC) group included 15 females and 13 males with a mean age of 56.4 (SD = 11.9) years. HC were included if they had no history of a mental disorder or a neurological disease. The study was carried out in accordance with The Code of Ethics of the World Medical Association (Declaration of Helsinki) for experiments involving humans. All participants gave written informed consent as approved by the local institutional authority (Health District Bruneck/South Tyrol‐Italy).

### 2.2 Structural Neuroimaging and Data Acquisition

Structural data were acquired in the Department of Radiology at the General Hospital Bruneck, South Tyrol, Italy, using an MRI system at 1.0 Tesla (Philips INTERA, Release 11, Best, The Netherlands). A FLAIR sequence was used with TR = 11 000 ms, TE = 140 ms, TI = 2800 ms, FA = 90°. Further, a 3D T1 gradient echo recalled (fast field echo, FFE) sequence was used with TR = 25 ms, TE = 6.9 ms; FOV = 230 mm [AP], 172 mm [RL]; resolution = 0.9 mm^3^; number of slices = 170. All images were visually inspected by an experienced clinical neuroradiologist (M.K.) to ensure the absence of severe motion artefacts [19].

### 2.3 White‐Matter Lesion Segmentation and Between‐Group Analyses

For WML segmentation, the LST‐AI toolbox was used [18], which is a deep learning‐based successor of the Lesion Segmentation Toolbox (LST) introduced by Schmidt and colleagues [20]. LST‐AI explicitly addresses the imbalance between WML and non‐lesioned white matter. It employs a composite loss function incorporating binary cross‐entropy and Tversky loss to improve segmentation of highly heterogeneous WML.

For data analysis, first the raw T1‐ and FLAIR‐images were converted into compressed NIfTI‐format (*.nii.gz) via dcm2niix, which is included in MRIcroGL (https://www.nitrc.org/projects/mricrogl; last visited 01/13/2025). Afterwards, LST‐AI (implemented in Ubuntu 22.04 LTS) was applied on each subject in CPU‐only mode and temporal files were stored to achieve segmented subject WML maps in MNI space for further processing in SPM12 (https://www.fil.ion.ucl.ac.uk/spm/software/spm12/; last visited 01/14/2025). During LST‐AI preprocessing, hd‐bet [21] and greedy [22] were applied for skull‐stripping and image registration, respectively. For between‐group comparisons, an SPM full factorial model was used based on the segmented WML maps in MNI space including group (HC, DI and non‐DI) as a factor. Age, sex and CPZ‐equivalents were considered as nuisance variables. We first considered the main effect of group, that is, HC versus all DD patients (contrast: HC 1, DI −0.5, non‐DI −0.5 and vice versa). Subsequently, we conducted comparisons between HC versus DI or non‐DI patients (contrast: HC 1, DI −1 and HC 1, non‐DI −1, and vice versa) and DI versus non‐DI patients (contrast: DI 1, non‐DI −1 and vice versa). A significance threshold of *p* < 0.005 (uncorrected at the voxel level, corrected for spatial extent using random field theory [minimum cluster size 147 voxels]) was used. Anatomical regions emerging from between‐group comparisons were labelled according to the Talairach Daemon (TD, http://www.talairach.org/daemon.html), using the ‘single point’ and subsequently the ‘nearest gray matter’ option to highlight the location of WML in close proximity to well‐known Brodmann areas (BA).

To be complete, in a complementary analysis, we tested the effect of diagnosis (i.e., DD independent of delusional content) by means of a design matrix that included all patients grouped together in a single category, that is, a two‐sample *t*‐test adjusted for age, sex and CPZ‐equivalents. Further, we also tested whether an adjustment for total intracranial volume (TIV) would have an impact on WMLL differences between the groups. For this purpose, TIV was calculated using T1‐weighted structural images and data processing algorithms provided by the Computational Anatomy Toolbox 12 (CAT12, https://neuro‐jena.github.io/cat/).

## 3 Results

### 3.1 Demographic and Clinical Data

The patient groups did not significantly differ from each other with respect to sex (all *p* > 0.05), but DI‐patients were older than HC (*p* < 0.001) and non‐DI‐patients (*p* = 0.008). HC and non‐DI‐patients did not significantly differ in age (*p* = 0.67). Disease duration in DI‐patients was shorter compared to the non‐DI group (*p* = 0.01). The patient groups did not differ in terms of CPZ equivalents (*p* = 0.033).

### 3.2 MRI Data

Considering the main effect of group, patients with DD (irrespective of content) showed higher WMLL in the left anterior cingulate gyrus (ACC), proximal to BA32. Increased WML in DD patients versus HC was found in bilateral superior frontal gyrus (SFG), proximal to BA8, right middle frontal gyrus (MFG) proximal to BA6, and left middle temporal gyrus (MTG) proximal to BA39, extending into superior parietal portions. These regions were also confirmed in a complementary analysis using a design matrix that included all patients grouped together in a single category.

**FIGURE 1:**
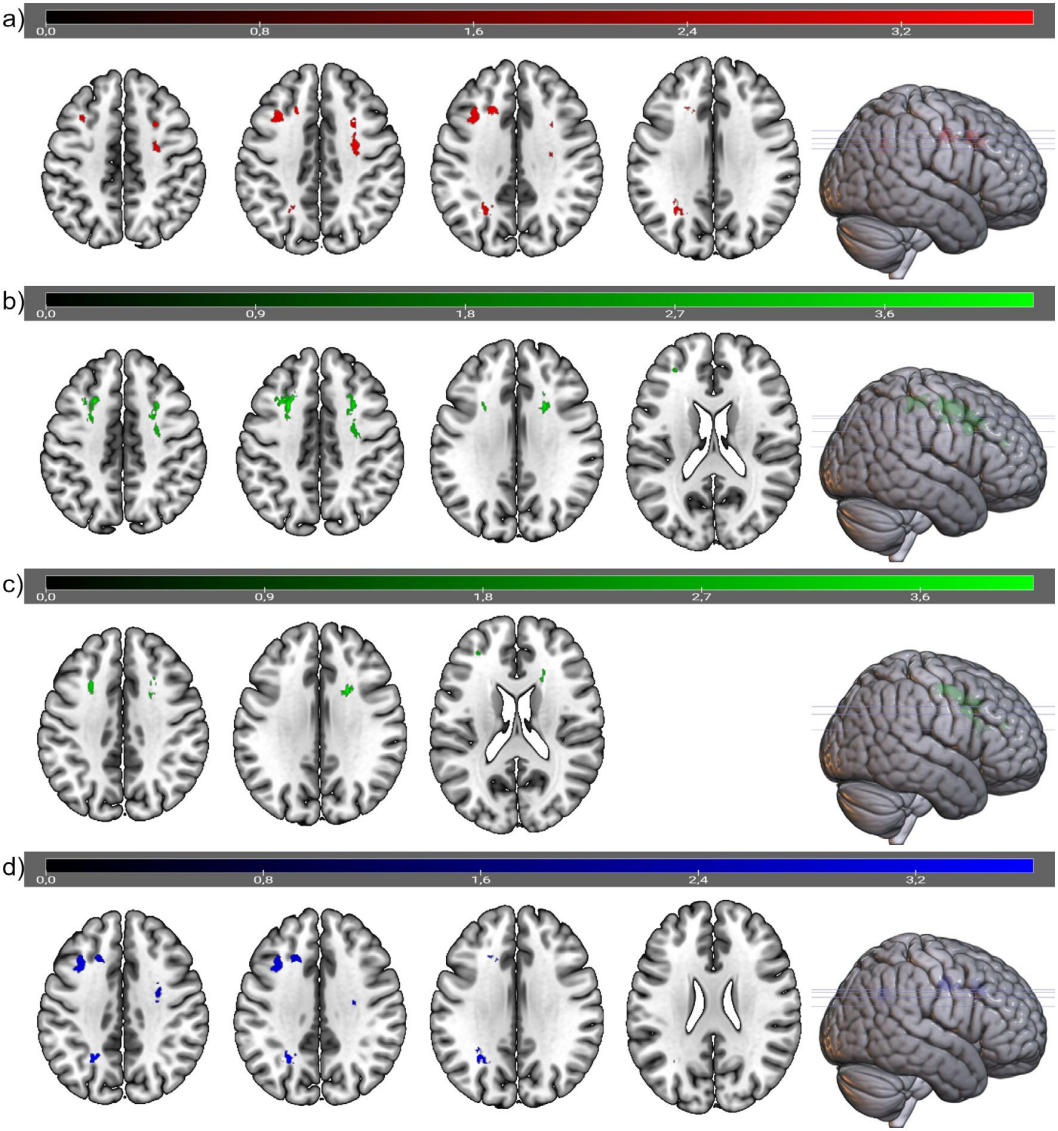
Spatial localization of WML differences between groups. Overlays of statistical maps on the spm152‐template in neurological convention (left on left). (a) DD patients > HC (red). (b) DI > HC (green). (c) DI > non‐DI (green). (d) non‐DI > HC (blue). Colour bars show *T*‐values; slices are in axial orientation at the z‐coordinates with maximum values of reported clusters; renderings show cluster localizations and slice positions (lines); all maps are thresholded at *p* < 0.005 with minimum cluster extent of 147 voxels. This figure was created using MRIcroGL and GIMP (https://www.gimp.org/; last visited 01/13/2025).

Considering the DD groups separately, patients with DI versus HC showed a higher WMLL in right MFG (BA6), right ACC (BA 32), left SFG (BA8), and left MFG proximal to BA10. WMLL in right ACC, left SFG, and left MFG was also higher in DI versus non‐DI patients. Eventually, non‐DI patients showed higher WMLL compared to HC in left and right ACC (BA 32 and BA 24), left MTG (BA39) and left MFG (in proximity to BA9) (see Figure 1 and Table 1). All stereotaxic coordinates and Z‐Scores are available upon request. In none of the contrasts was WMLL higher in HC compared to the patient groups. The integration of TIV as an additional covariate in the factorial models resulted in only marginal differences in cluster size. Despite these slight variations, the analysis confirmed the findings of the original full factorial model.

**TABLE 1:** Comparison of regional white matter lesion load (WMLL) across patient groups with delusional disorder (DD), including individuals with delusional infestation (DI) and without DI (non‐DI), and healthy controls (HC). Regions emerging from between‐group comparisons were identified based on their proximity to Brodmann Areas (BA) and further described in terms of their anatomical location and associated cognitive or affective functions (see main text for further details).

Comparison	BA (approx.)	Anatomical region	Ascribed functions
DI > HC	BA6	Right middle frontal gyrus	Premotor cortex; motor planning; sensorimotor integration
	BA8	Left superior frontal gyrus	Frontal eye fields: visual attention, attentional bias toward bodily sensations
	BA10	Left middle frontal gyrus (rostral portion)	Metacognition; self‐monitoring; belief evaluation
	BA 32	Right anterior cingulate cortex	Error/conflict monitoring; emotional salience; prediction error processing
non‐DI > HC	BA24, BA32	Bilateral anterior cingulate cortex	Regulation of threat/anxiety; conflict monitoring; hypervigilance (paranoid content)
	BA39	Left middle temporal gyrus	Theory of mind; self‐other distinction; misattribution of intent
	BA9	Left middle frontal gyrus	Executive function; cognitive control; integration of external and internal stimuli
DI > non‐DI	BA8	Left superior frontal gyrus	Visual attention; over‐focusing on bodily cues
	BA10	Left middle frontal gyrus (rostral portion)	Impaired self‐referential processing and belief evaluation
	BA32	Right anterior cingulate cortex	Amplified salience of bodily sensations; failure of error correction

## 4 Discussion

This structural magnetic resonance imaging (MRI) study investigated white matter lesion load (WMLL) in patients with delusional infestation (DI) versus non‐DI. Two main findings emerged: 1. Regions with higher WMLL in both DI and non‐DI patients compared to HC included the anterior cingulate cortex (ACC) and middle frontal gyrus (MFG), 2. Regions with higher WMLL in DI patients versus both HC and non‐DI patients included further regions of the frontal lobe, particularly in superior frontal gyrus (SFG) and MFG. To facilitate the interdisciplinary dialogue between clinicians, neuroradiologists and neuroscientists alike, we will elaborate on the significance of these findings on a region‐by‐region basis and by contrasting findings in DI versus non‐DI.

An important component of DI pathophysiology seems to involve the premotor cortex and supplementary motor area, that is, regions involved in motor planning and sensorimotor integration [23, 24]. In somatic delusions, a dysfunction of these regions may amplify normal bodily sensations, such as minor tingling, itching or muscle twitches, and subsequently lead to their misinterpretation as evidence of bodily abnormalities (e.g., parasites crawling under the skin) [8, 9]. This can occur due to deficient motor‐related sensory feedback, where such a dysfunction may cause a distortion in the way sensory signals are integrated with motor awareness [25], contributing to heightened focus on bodily sensations [2]. In non‐somatic delusions, the role of premotor/supplementary motor could be less prominent. This notion is also consistent with clinical observations; non‐somatic delusions primarily revolve around external events, intentions or abstract beliefs rather than bodily sensations, thereby engaging the sensorimotor integration process to a lesser extent.

Brodmann Area (BA) 8, encompassing the frontal eye fields (FEF), is responsible for visual attention, gaze control and orienting focus toward salient stimuli [26, 27]. In somatic delusions, dysfunction in BA8 may lead to an excessive attentional bias toward bodily sensations or physical anomalies. This notion is in good agreement with the typical clinical picture of DI, since affected patients often describe meticulously observing their body (e.g., skin lesions and perceived infestations) [28, 29, 30]. Abnormalities of higher order visual processing areas could reflect the inability to disengage from these bodily cues, heightening their significance. This over‐focusing could also perpetuate the preoccupation with perceived somatic abnormalities. In contrast, non‐somatic delusions are less tied to internal bodily cues and rely more on external stimuli and related cognitive processes. It is possible that in such cases, contributions from visual areas are more likely to be related to heightened vigilance toward external stimuli, for example, suspicious movements, speech or other ‘signs’ in one's peripersonal space [31].

The rostral portions of the SFG and the MFG, as assigned to BA10, predominantly govern action monitoring, metacognition and self‐referential thinking [32, 33, 34, 35]. In somatic delusions, dysfunction of such processes may impair the ability to evaluate the plausibility of bodily sensations, leading to distorted self‐monitoring. For instance, minor or normal somatic sensations (e.g., skin irritation and heartbeat awareness) are interpreted as pathological due to impaired cognitive integration and failure to suppress irrelevant internal signals [12]. Dysfunction of rostral areas of the MFG also could weaken the ability to generate alternative, rational explanations, solidifying the delusion. In non‐somatic delusions, rostral prefrontal cortex dysfunction similarly may impair reality testing but focuses on external events or beliefs, such as misinterpreting others' intentions in paranoid delusions [2, 3, 36]. While both types of delusions may share rostral prefrontal cortex impairment, the specific content (internal body‐focused versus external world‐focused) depends on where attention and cognitive resources are directed.

The anterior cingulate cortex (ACC) plays a crucial role in executive functions such as action and conflict monitoring [37, 38]. Furthermore, the involvement of the ACC in error monitoring is well‐established and has been integrated into broader theoretical models of delusion formation, which emphasise the role of prediction errors [2, 3]. In somatic delusions, ACC dysfunction could be related to an amplification of emotional salience attributed to bodily sensations, that is, normally benign sensations may evoke exaggerated emotional responses, leading to distress and a heightened focus on perceived bodily abnormalities. This dysfunction disrupts error monitoring [38], preventing the patient from questioning the irrationality of their beliefs and reinforcing their fixation on somatic sensations [2]. In non‐somatic delusions, it is conceivable that effective that similar processes occur, that is, the amplification of the emotional salient external events. Given the very distinct delusional content, it is possible that in somatic delusions, ACC contributions are more tightly linked to internal bodily signals and their emotional appraisal, whereas in non‐somatic delusions, focus lies on external cues.

In terms of ACC involvement in DD, it is noteworthy that WML close to BA24 were detected in non‐DI patients in contrast to individuals with DI. This portion of the brain region is crucially involved in regulation of threat and anxiety [39], which are hallmarks of persecutory delusions. This is also consistent with the clinical picture of paranoid or persecutory delusions, as patients very often experience excessive anxiety, fear and hypervigilance, as a consequence of constant threat from others [1, 40]. Further, it is important to note that unlike DI, non‐DI patients also showed higher WMLL in proximity to parietal cortex BA39, a region involved in theory of mind and self‐referential processes [41]. This said, abnormalities found in this part of the brain can lead to abnormal processing of both external and internal inputs, which may contribute to misattributions of intent or agency, as much as they can contribute to an aberrant ability to infer others' intentions, emotions and perspectives [42, 43]. Dysfunction here may impair this ability, leading to misinterpretations of social cues and an excessive tendency to attribute malevolence to others, which is a clinical hallmark feature of paranoid delusions.

Beyond the regional distribution of WMLL, it is critical to consider the neurobiological implications of such lesions in the context of DD. WML are increasingly conceptualised not merely as structural abnormalities, but as markers of disrupted brain network integrity, particularly affecting long‐range cortico‐cortical and cortico‐subcortical connectivity [44]. Such disruptions can undermine the brain's capacity for efficient communication between regions involved in cognitive control, salience detection and self‐monitoring [45]. In the context of delusional psychopathology, impaired structural connectivity may give rise to the formation of false beliefs through mechanisms such as aberrant salience attribution, reduced error monitoring and diminished cognitive flexibility [2, 12, 46]. This could be particularly relevant in somatic DD, where disrupted integration between sensorimotor, attentional and metacognitive systems could foster misinterpretations of bodily sensations and hinder the updating of implausible beliefs [11, 12]. Hence, WML may serve as a neurobiological substrate that promotes the persistence of delusional ideation by compromising the hierarchical organisation of predictive processing networks [2].

Furthermore, the role of aging needs to be considered in the etiopathogenesis of WML and its interaction with delusional symptoms. Age‐related cerebrovascular changes are a major contributor to the accumulation of WML, with increasing evidence pointing to their association with neurocognitive decline and altered affective processing [44, 47]. Concurrently, healthy aging is accompanied by a gradual decline in dopaminergic transmission [48] and a reorganisation of functional brain networks [49], including frontoparietal and cortical midline networks—both of which are implicated in belief evaluation and self‐referential processing [3, 46]. The convergence of WML‐related disconnection and age‐associated neurobiological alterations may thus constitute a ‘second hit’ in vulnerable individuals [17], lowering the threshold for the emergence or persistence of delusional ideas. Importantly, the interaction between vascular pathology, dopaminergic decline [11], and network‐level dysfunction could provide a plausible framework for understanding why DD, particularly late‐onset subtypes, may be more prevalent or persistent in older populations [5].

Potential limitations of this study include the modest sample sizes and the use of psychotropic medication in the patient samples. Age and disease duration need to be considered as potentially relevant variables as well, since the non‐DI group was significantly younger compared to both DI patients and HC, and since non‐DI patients presented with longer disease duration. Moreover, the lack of psychosis severity measures limits the ability to examine associations between the extent of delusional severity and WMLL. Eventually, the limited MRI field strength could yield an increased risk of false negative findings due to the lower signal‐to‐noise ratio compared with higher field strengths, for example, 3 T or higher.

## 5 Conclusion

Keeping this study's limitations in mind, we provide first controlled evidence for distinct patterns of WMLL in patients with DI versus non‐DI. Shared impairments across that patient groups, regardless of delusional content, reflect core deficits in attention, reasoning and emotional regulation. The data underscore how a dysfunction across areas controlling sensorimotor integration process could drive the internal bodily focus of somatic delusions versus the externalised cognitive and affective distortions seen in non‐somatic DD. Such insights may also guide clinical differential diagnosis and could also increase awareness of neuroradiologists for pathophysiological relevant brain imaging findings.

## Ethics Statement

The study was carried out in accordance with The Code of Ethics of the World Medical Association (Declaration of Helsinki) for experiments involving humans. All participants gave written informed consent as approved by the local institutional authority (Health District Bruneck/South Tyrol‐Italy).

## Conflicts of Interest

The authors declare no conflicts of interest.

## Data Availability

Data availability requests are handled by the Health District Bruneck/South Tyrol‐Italy, following national and EU data protection regulations.
